# Phase I safety data of lenalidomide, bortezomib, dexamethasone, and elotuzumab as induction therapy for newly diagnosed symptomatic multiple myeloma: SWOG S1211

**DOI:** 10.1038/bcj.2015.62

**Published:** 2015-08-07

**Authors:** S Z Usmani, R Sexton, S Ailawadhi, J J Shah, J Valent, M Rosenzweig, B Lipe, J A Zonder, S Fredette, B Durie, A Hoering, B Bartlett, R Z Orlowski

**Affiliations:** 1 Levine Cancer Institute/Carolinas HealthCare System, Charlotte, NC, USA; 2 Cancer Research and Biostatistics, Seattle, WA, USA; 3 Mayo Clinic, Jacksonville, FL, USA; 4 MD Anderson Cancer Center, Houston, TX, USA; 5 Taussig Cancer Institute, Cleveland Clinic, Cleveland, OH, USA; 6 City of Hope, Los Angeles, CA, USA; 7 University of Kansas Cancer Center, Westwood, KS, USA; 8 Karmanos Cancer Institute, Detroit, MI, USA; 9 SWOG Operations, San Antonio, TX, USA; 10 Cedar-Sinai Medical Center, Los Angeles, CA, USA; 11 Bristol-Myers Squibb, Princeton, NJ, USA

Novel drugs including immunomodulatory agents and proteasome inhibitors have improved outcomes in plasma cell dyscrasias, but high-risk multiple myeloma (HRMM) retains a poor prognosis and remains a therapeutic challenge. Even with aggressive Total Therapy approaches, poor genomic risk patients have a 2-year event-free survival of ~50%.^1^ An intergroup, randomized trial was designed to evaluate the efficacy of adding elotuzumab (Elo) into the front line for HRMM, comparing lenalidomide, bortezomib and dexamethasone (RVd) with or without Elo. This agent is a humanized monoclonal antibody to SLAMF7, a cell surface glycoprotein on myeloma cells but with limited expression on normal cells,^2^ and early clinical studies of Elo has shown promise. In a phase I of Elo/bortezomib, the overall response rate (ORR), including partial response (PR) or better, was 48%.^3^ In Phase Ib^4^ and II^5^ studies of Elo/Rd, the ORR was 82% and 92%, respectively, for patients treated with Elo at 10 mg/kg. For all Elo studies, adverse events (AEs) were primarily infusion related and manageable using adequate premedication. Though limited, the data available suggest these Elo-based combinations have comparable response rates in high-risk and standard-risk relapsed and/or refractory patients, providing a rationale for its incorporation into front-line HRMM therapy.

The current report focuses on the Phase I portion of the randomized study, whose objective was to, for the first time, determine the maximum tolerated dose (MTD) of the four-drug RVd-Elo regimen. All newly diagnosed patients with symptomatic myeloma regardless of risk were eligible for this portion, which was conducted through SWOG centers. Importantly, the randomized Phase II intergroup effort will focus solely on HRMM, defined by one of the following: poor risk genomics by the Arkansas 70-gene model; or either translocation (14;16), (14;20), 1q21 amplification or deletion 17p by florescent *in situ* hybridization; or primary plasma cell leukemia; or serum lactate dehydrogenase >twice the upper limit of normal. Treatment consisted of induction for eight cycles with RVd-Elo (lenalidomide 25 mg orally, days 1–14 of every 21-day cycle; bortezomib 1.3 mg/m^2^ subcutaneously, days 1, 4, 8 and 11; dexamethasone 20 mg orally, days 1, 2, 4, 5, 8, 9, 11 and 12; elotuzumab 10 mg/kg intravenously, days 1, 8 and 15 of cycles 1–2, then days 1 and 11 of cycles 3–8). This was followed by dose-attenuated RVd-Elo maintenance (lenalidomide 15 mg orally, days 1–21 of every 28-day cycle; bortezomib 1.0 mg/m^2^ subcutaneously, days 1, 8 and 15; dexamethasone 12 mg orally, days 1, 8 and 15; elotuzumab 10 mg/kg intravenously, days 1 and 15) until disease relapse, progression or intolerance. AEs were recorded as per the Common Terminology Criteria for Adverse Events, v4.0.

Eight newly diagnosed patients were enrolled to the Phase I portion of the trial, among whom six received treatment and were evaluable for dose-limiting toxicities (DLTs) during cycle 1, as per protocol. The median patient age was 67 years (range: 56–79), hemoglobin was <10 g/dl in 50%, and creatinine was <2 mg/dl in all patients. International Staging System stage distribution was 17% (I), 33% (II) and 50% (III). The most common AEs (Table 1) for the study to date have been fatigue (100%), peripheral sensory neuropathy (83%), edema (83%), lymphopenia (66%) and leukopenia (50%). One DLT (grade 4 lymphopenia) was observed. The peripheral sensory neuropathy rates are similar to the Richardson *et al.*^6^ experience, where 80% subjects developed these symptoms, with 27% subjects developing grade 3 or above. All six patients have completed eight cycles of induction and five have completed at least four maintenance cycles. Overall median days on therapy per cycle were 13 days during induction and 20 days during maintenance. Dose adjustments were made in 83% of patients for bortezomib, 83% for lenalidomide, 33% for dexamethasone and 50% for elotuzumab. The elotuzumab adjustments included two dose delays (one due to AE and one per study chair recommendation) and one dose withholding due to AE. Efficacy data will be released when the Phase II randomized study findings are mature.

In summary, RVd-Elo is a feasible regimen for newly diagnosed myeloma patients without major additive AE/SAE beyond what is already known about RVd.^6^ This is the first report of the only Phase I experience combining the triple-drug regimen RVd with the monoclonal antibody Elo for newly diagnosed myeloma, and has identified a dose for further study. These data have informed the SWOG 1211 Phase II dosing, as well as other trials for transplant-eligible patients.

## Figures and Tables

**Table 1:** Adverse events

*Event*	*Total (%)*	*Grade 3 (%)*	*Grade 4 (%)*
ALT increased	1 (16%)	—	—
AST increased	1 (16%)		
Abdominal pain	2 (33%)	—	—
Alkaline phosphatase increased	2 (33%)		
Anorexia	2 (33%)	—	—
Anxiety	1 (16%)	—	—
Arthralgia	1 (16%)	—	—
Back pain	2 (33%)	—	—
Bloating	1 (16%)	—	—
Blurred vision	1 (16%)	—	—
Bruising	1 (16%)	—	—
Constipation	3 (50%)	—	—
Creatinine increased	1 (16%)	—	—
Diarrhea	2 (33%)	—	—
Dry skin	1 (16%)	—	—
Dyspnea	2 (33%)	—	—
Ear/labyrinth disorders	1 (16%)		
Edema limbs	5 (83%)	—	—
Erythema multiforme	1 (16%)	—	—
Flu like symptoms	1 (16%)	—	—
GERD	1 (16%)	—	—
Heart failure	1 (16%)	—	—
Hypertension	1 (16%)	—	—
Hypokalemia	1 (16%)	—	—
Immune system disorders	2 (33%)		
Infections	1 (16%)	—	—
Injection site reaction	1 (16%)	—	—
Insomnia	2 (33%)	—	—
Irritability	1 (16%)	—	—
Leukocytosis	1 (16%)	1 (16%)	—
Lymphocyte count decreased	4 (66%)	—	1 (16%)
Muscle weakness lower limb	1 (16%)	—	—
Nausea	2 (33%)	—	—
Nervous system disorders	1 (16%)	1 (16%)	
Vomiting	3 (50%)	—	—
Neutrophil count decreased	1 (16%)	1 (16%)	—
Painful neuropathy	1 (16%)	—	—
Peripheral sensory neuropathy	5 (83%)	2 (33%)	—
Platelet count decreased	3 (50%)	1 (16%)	—
Rash maculo-papular	1 (16%)	—	—
Sinus bradycardia	1 (16%)		
Skin hyperpigmentation	1 (16%)		
Skin/subcutaneous tissue disorder	1 (16%)		
Thromboembolic event	1 (16%)	—	—
Tinnitus	1 (16%)	—	—
Upper respiratory infection	1 (16%)	—	—
Weight loss	2 (33%)	—	—
White blood cell decreased	3 (50%)	—	—
Fatigue	6 (100%)	—	—

## Notes

SZU is a consultant for Celgene, Millennium, Onyx and Sanofi; RZO is a consultant and has received research funding for ArrayBioPharma, Bristol-Myers Squibb, Celgene, Janssen, Millennium, Onyx and Pharmacyclics; SA received honorarium from Millennium. JS is a consultant to Celgene, Millennium, Onyx and Novartis and has received research funding for ArrayBioPharma, Celgene, Millennium, Onyx and Novartis. JV has received speaking honoraria from Celgene and Millennium. The remaining authors declare no conflict of interest.

## References

[bib1] Nair B van Rhee F Shaughnessy JD Jr Anaissie E Szymonifka J Hoering A Superior results of Total Therapy 3 (2003-33) in gene expression profiling-defined low-risk multiple myeloma confirmed in subsequent trial 2006-66 with VRD maintenance Blood 2010 115 4168 4173 20124509 10.1182/blood-2009-11-255620 PMC2879104

[bib2] Tai YT Dillon M Song W Leiba M Li XF Burger P Anti-CS1 humanized monoclonal antibody HuLuc63 inhibits myeloma cell adhesion and induces antibody-dependent cellular cytotoxicity in the bone marrow milieu Blood 2008 112 1329 1337 17906076 10.1182/blood-2007-08-107292 PMC2515112

[bib3] Jakubowiak AJ Benson DM Bensinger W Seigel DS Zimmerman TM Mohrbacher A Elotuzumab in combination with bortezomib in patients with relapsed/ refractory multiple myeloma: updated results of a phase 1 study Blood 2010 116 302320664053

[bib4] Lonial S Vij R Harousseau JL Facon T Moreau P Mazumder A Elotuzumab in combination with lenalidomide and low-dose dexamethasone in relapsed or refractory multiple myeloma J Clin Oncol 2012 30 1953 1959 22547589 10.1200/JCO.2011.37.2649

[bib5] Richardson PG Jagannath S Moreau P Jakubowiak A Raab MS Facon T A Phase 2 study of elotuzumab (Elo) in combination with lenalidomide and low-dose dexamethasone (Ld) in patients (pts) with relapsed/refractory multiple myeloma (R/R MM): updated results Blood 2012 118 202

[bib6] Richardson PG Weller E Lonial S Jakubowiak AJ Jagannath S Raje NS Lenalidomide, bortezomib, and dexamethasone combination therapy in patients with newly diagnosed multiple myeloma Blood 2010 116 679 686 20385792 10.1182/blood-2010-02-268862 PMC3324254

